# A–X⋯σ Interactions—Halogen Bonds with σ-Electrons as the Lewis Base Centre

**DOI:** 10.3390/molecules26175175

**Published:** 2021-08-26

**Authors:** Sławomir J. Grabowski

**Affiliations:** 1 Polimero eta Material Aurreratuak: Fisika, Kimika eta Teknologia, Kimika Fakultatea, Euskal Herriko Unibertsitatea UPV/EHU & Donostia International Physics Center (DIPC) PK 1072, 20080 Donostia, Spain; s.grabowski@ikerbasque.org; 2 IKERBASQUE, Basque Foundation for Science, 48011 Bilbao, Spain

**Keywords:** halogen bond, σ-hole, dihydrogen, cycloalkanes, σ-electrons, crystal structures

## Abstract

CCSD(T)/aug-cc-pVTZ//ωB97XD/aug-cc-pVTZ calculations were performed for halogen-bonded complexes. Here, the molecular hydrogen, cyclopropane, cyclobutane and cyclopentane act as Lewis base units that interact through the electrons of the H–H or C–C σ-bond. The FCCH, ClCCH, BrCCH and ICCH species, as well as the F_2_, Cl_2_, Br_2_ and I_2_ molecular halogens, act as Lewis acid units in these complexes, interacting through the σ-hole localised at the halogen centre. The Quantum Theory of Atoms in Molecules (QTAIM), the Natural Bond Orbital (NBO) and the Energy Decomposition Analysis (EDA) approaches were applied to analyse these aforementioned complexes. These complexes may be classified as linked by A–X···σ halogen bonds, where A = C, X (halogen). However, distinct properties of these halogen bonds are observed that depend partly on the kind of electron donor: dihydrogen, cyclopropane, or another cycloalkane. Examples of similar interactions that occur in crystals are presented; Cambridge Structural Database (CSD) searches were carried out to find species linked by the A–X···σ halogen bonds.

## 1. Introduction

Halogen bonds have been analysed in numerous theoretical and experimental studies; they are an interaction where, surprisingly, an electronegative halogen centre plays the role of a Lewis acid site [1,2,3,4,5,6,7,8,9,10,11,12,13]. The nature of halogen bonds is the subject of numerous discussions and polemics. It was supposed that this interaction results from a positive net atomic charge of halogen that is often observed in numerous systems, especially for heavier halogens such as bromine and iodine. However, the interactions of halogens possessing negative atomic charges and acting as the Lewis acid centres have also been detected.

Various explanations of the formation of halogen bonds are related to the anisotropy of the electron charge distribution at the halogen centre [14]. Early studies proposed the idea of a direction-dependent van der Waals radius [15]. It was explained in more recent studies that for a carbon–halogen bond, C–X, ellipsoidal-shaped electron charge distribution around halogen centre often occurs [14,16]. The longest semi-axis of this ellipsoid is approximately perpendicular to the C–X bond, whereas the shortest semi-axis is consistent with the direction of this bond. This explains why halogen centres are often characterised by a dual role. They can act as Lewis acids in the extension of C–X bonds and they can act as Lewis bases in the directions approximately perpendicular to these bonds [17]. It has been described in several studies that sometimes other bonds, not only C–X, are characterised by the dual character of halogen centres. For example, this was analysed for Si-X bonds [18].

The dual character of halogen centres mentioned above is related to the σ-hole concept [3,4,5,6,7], which states that in the extension of C–X bonds (and sometimes of other A–X bonds) electron charge depletion occurs (σ-hole). This is connected with the increased electrostatic potential, EP, at this site, up to the positive values, and with the increase in Lewis acid properties of the halogen centre. It is interesting that the concept of the σ-hole [3,4,5,6,7,19,20] and of the π-hole [19,20] explains the formation of other interactions, not only halogen bonds. Interactions such as aerogen, chalcogen, pnicogen, and tetrel bonds have been described in terms of the above concept [3,4,5,6,7,19,20,21,22]; these interactions concern the 18th, 16th, 15th and 14th groups of elements, respectively.

The triel elements (13th group) may also act as the Lewis acid centres [23,24], mainly through π-holes characterised by the positive EP. However, σ-holes at the triel centres are also possible [24]. The Lewis acid properties of triel elements result from their electron structures [25,26,27,28,29] because triel elements are often electron-deficient in planar compounds; they have six electrons in their outer shells. The interaction of these centres with electron-donating ligands leads to the complement of the electron octet structures [30]. The σ-hole and π-hole concept explains the occurrence of areas of positive electrostatic potential in triel centres, and therefore, their electrostatic properties, whereas additional insight into their electron structures explains the electron charge shifts that occur for triel-bonded systems.

The σ-hole and π-hole bonds mentioned above have been analysed in various studies and compared with hydrogen bonds. It was pointed out that even hydrogen bonds could be classified as σ-hole bonds [6,19]. Therefore, it is worth mentioning here that various classes of hydrogen bond interaction have been specified in numerous studies. One such classification is based on a type of proton acceptor (electron donor). It was stated that, apart from the typical A–H···B hydrogen bonds characterised by the single-centre proton acceptor (B), and the A–H···π interactions with multi-centre proton acceptors related to π-electrons, there are also A–H···H–B dihydrogen bonds where the H–B σ-bonds may be considered as the proton acceptors [31,32]. Hence, the latter interactions may be treated as the third kind of hydrogen bond and designated as A–H···σ interactions. However, it was justified that A–H···H–B dihydrogen bonds are an interaction with the single proton-accepting centre that is a negatively charged hydrogen atom connected with the B centre [33]. In this way, in the dihydrogen bond, two H atoms of opposite charges are in contact, whereas the A–H···σ interaction is characterised by other properties [33]. The bond of molecular hydrogen can act as the Lewis base site in A–H···σ interactions [33,34]; C–C bonds in cycloalkanes or other single σ-bonds also can act as electron donors [35].

**Scheme 1:**
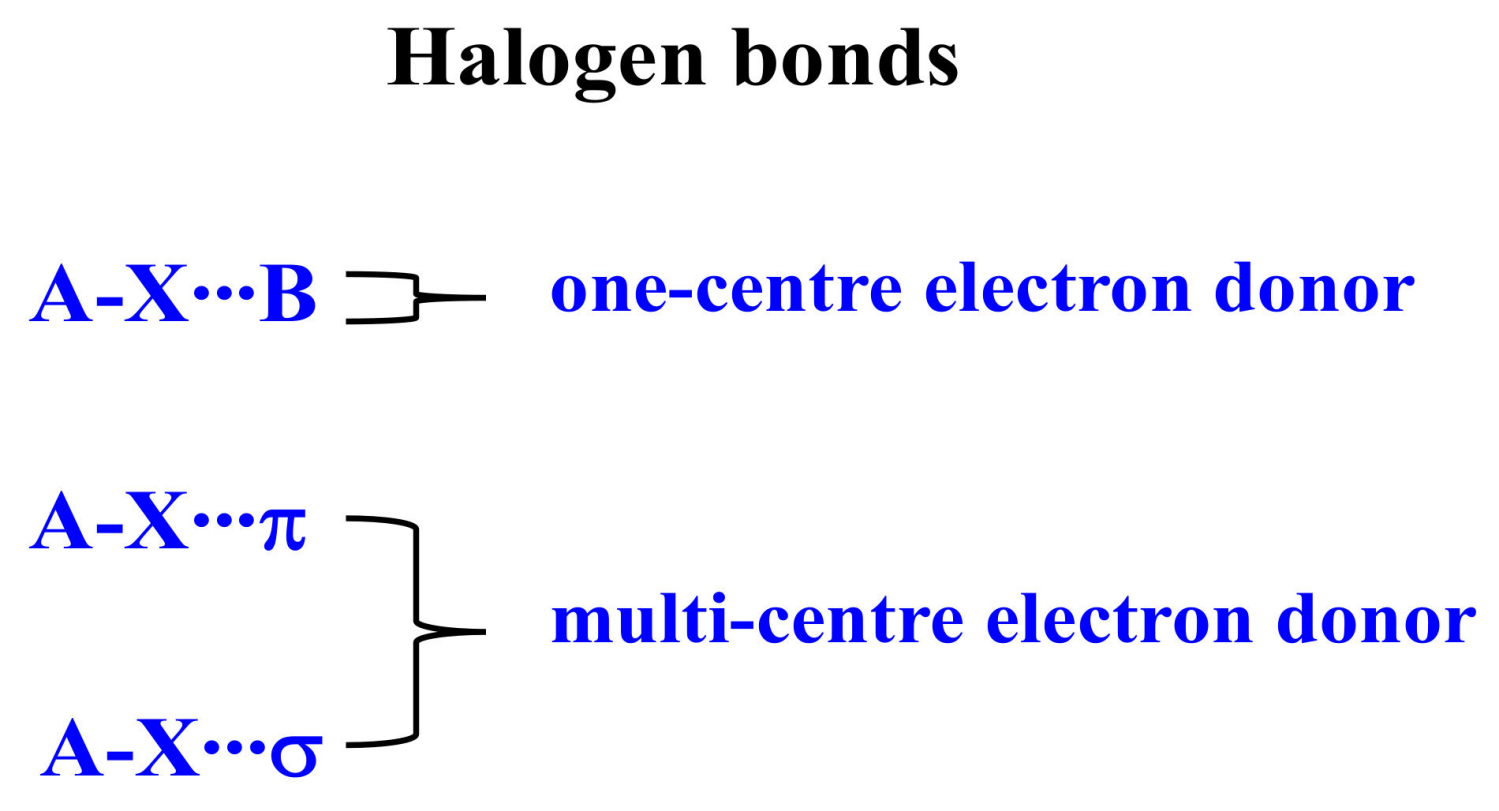
Types of halogen bond.

It was also discussed that molecular hydrogen may play a role of the Lewis base unit in other interactions such as the pnicogen, tetrel or triel bonds [36]. The latter topic is very important because it is related to studies concerning hydrogen storage materials [37,38,39]. A similar situation occurs for halogen bonds where one-centre or multi-centre Lewis base sites are observed [2]. Double or triple C–C bonds, as well as aromatic systems, may act as such electron donor sites. There are also halogen bond interactions with the σ-bonds acting as the electron donors [36,40]. Hence, the following types of halogen bonds may be specified: A–X···B, A–X···π, and A–X···σ (Scheme 1). However, the latter interactions have been discussed only marginally in the literature and, to the best of the author’s knowledge, only with molecular hydrogen acting as the Lewis base unit. This explains why the aim of this study was to characterise the broader range of A–X···σ interactions with not only dihydrogen as the electron donor, but also with cycloalkanes that act as the Lewis base units.

## 2. Results and Discussions

### 2.1. Examples of Crystal Structures

**Scheme 2:**
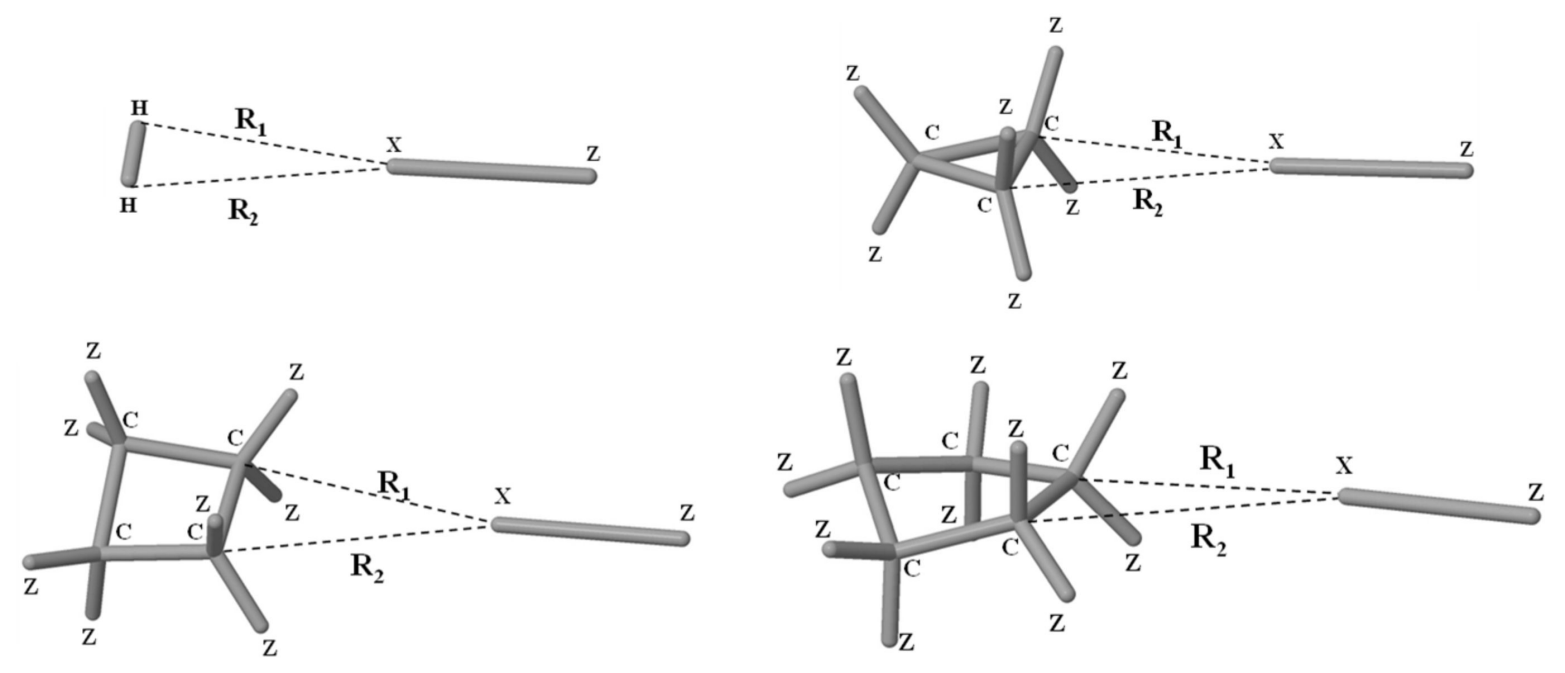
Four CSD searches characterised by dihydrogen and different cycloalkane rings as electron donors. Z designates any atom which may be further connected with greater fragments; X is the monovalent halogen centre. R_1_ and R_2_ are distances between the halogen centre and the H atoms or C atoms of the σ-bond playing a role in the Lewis base site.

Searches through the Cambridge Structural Database, CSD [41,42] (February, 2021 release), were performed to find crystal structures containing a halogen centre in contact with the σ-bond that plays a role in the Lewis base site. The following Lewis base units containing such bonds were taken into account: dihydrogen, and species containing rings of cyclopropane, cyclobutane, and cyclopentane. Hence, four CSD searches were performed for the above-mentioned sub-units of the complexes. The following criteria for these searches were applied: 3D coordinates determined, no disordered structures, no errors, no polymeric structures, an R-factor of less than or equal to 10%, and only single crystal structures. The searches concerned structures with intermolecular X⋯H distances or intermolecular X⋯C distances related to the A–X···σ interactions that were shorter than the corresponding sum of van der Waals radii (the R_1_ and R_2_ distances—see Scheme 2). The radii proposed by Bondi [43] that were inserted into the CSD set of codes were applied here. The X⋯H and X⋯C distances refer to the H–H and C–C σ-bonds, respectively, that play a role of the Lewis base sites. Scheme 2 shows systems that were searched through the CSD. One can see that for the four searches mentioned above, the X monovalent halogen centre connected with any atom (Z) which may in turn have been connected with any molecular fragment was taken into account. The same concerns the Z-substituents of cycloalkane rings which may be connected with other molecular fragments.

**Scheme 3:**
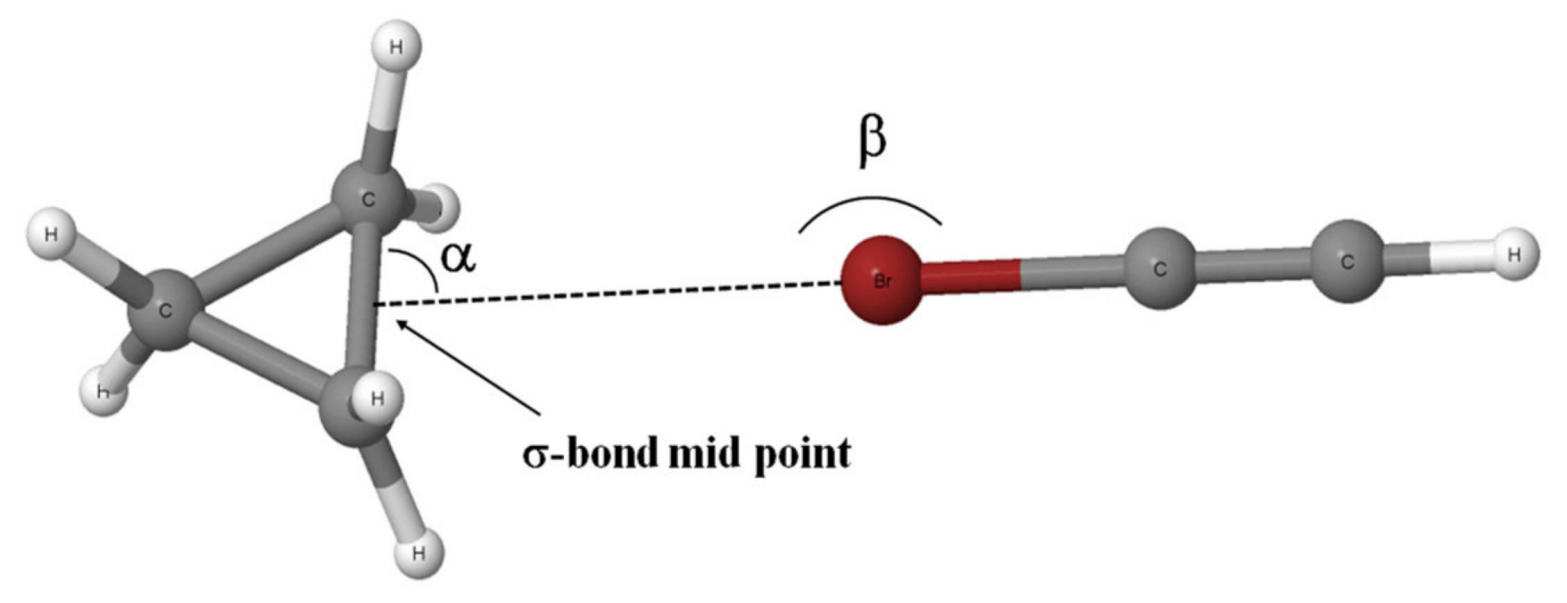
The definition α and β angles: the example of the C_3_H_6_···BrCCH complex.

Table 1 presents refcodes of crystal structures resulting from CSD searches, and the following data concerning these structures. Maximum estimated standard deviations (e.s.ds) of the bonds partly inform the accuracy of the crystal structures analysed, the types of A–X bonds that are involved in A–X···σ interactions, the X⋯σ distance between the halogen centre and the H–H or C–C bond midpoint, and the α and β angles which are defined in Scheme 3. The latter angles, equal to 90° and 180°, respectively, occur for an ideal T-shaped structural fragment containing the H–H or C–C bond and the A–X bond.

The first search on complexes of dihydrogen led to the finding of four structures. Three cases concern different refinements of the same crystal structure, (η^5^-pentamethyl-cyclopentadienyl)-(dihydrogen)-dihydrido- triphenylphosphine—osmium (iv) tetrafluoroborate (NOLJIH, NOLJIH01 and NOLJIH02 refcodes). The fourth case is the structure of (dihydrogen)—cis—dihydrido—(η^5^-pentamethylcyclopentadienyl)—(triphenylarsine)—osmium (iv) tetrafluoroborate (XAXMIT refcode). In these crystal structures, the dihydrogen is attached to the osmium centre and the BF_4_^−^ anion is very close to the dihydrogen. The F⋯σ (H–H midpoint) distances that may have corresponded to the halogen bonds are in a range from 2.43 to 2.54 Å. However, the existence of such local stabilising interactions seems to be controversial because the fluorine is a negatively charged centre directed to a negatively charged σ-bond; in addition, the β angles (Scheme 3) are rather far from 180°. The β angle closest to 180° would be the typical angle for the σ-hole bond-type interaction, as occurs for the halogen bond. It is interesting that all the A–X⋯σ arrangements presented in Table 1 that contain dihydrogen are B-F⋯σ contacts of the BF_4_^−^ anion. However, the consideration of the latter anion as a potential Lewis acid unit is rather unlikely. It seems that the molecular hydrogen is attached to the osmium centre. The nature of interactions between the molecular hydrogen and the transition metals has been discussed in numerous studies [44,45,46]; this subject is not covered in this study.

**Figure 1:**
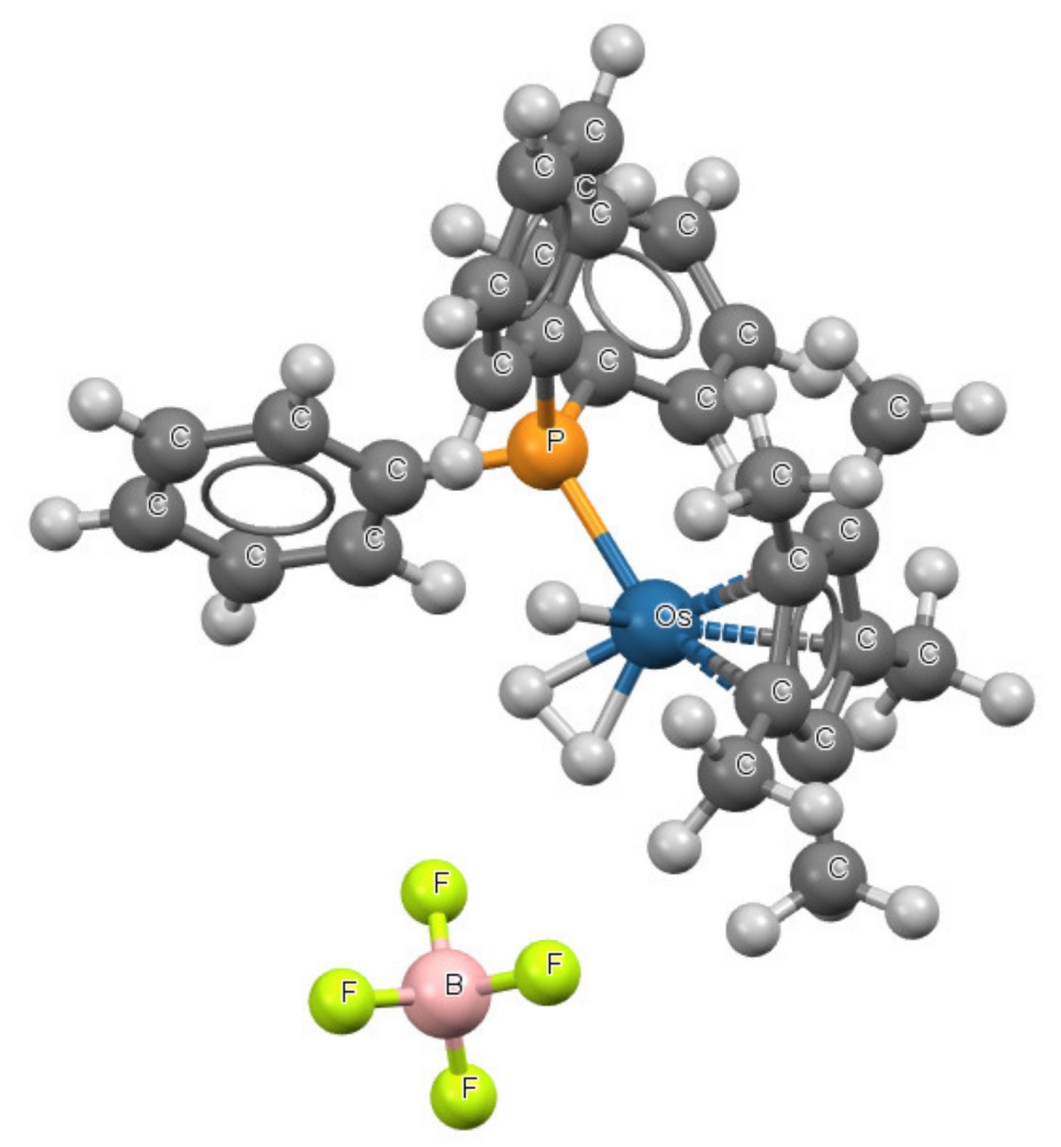
The fragment of the crystal structure of (η^5^-pentamethyl-cyclopentadienyl)-(dihydrogen)-dihydrido-triphenylphosphine-osmium (iv) tetrafluoroborate (NOLJIH refcode). Small grey circles correspond to H-atoms.

Figure 1 shows a fragment of the NOLJIH crystal structure where such a situation is observed; the H_2_ molecule is attached to the Os centre close to the BF_4_^−^ anion. The latter structure is the only neutron diffraction, ND, crystal structure (i.e., resolved by the ND method [47,48]) among those collected in Table 1; all other crystal structures were resolved by the X-ray diffraction methods. There are numerous crystal structures with the dihydrogen attached to the transition metal centre, and such arrangements are very important in issues related to hydrogen storage materials. This occurs for the dihydrogen structures discussed here where the BF_4_^−^ species are also located in the proximity of molecular hydrogen.

In a recent study, the molecular surface of the BF_4_^−^ species was discussed (MP2/aug-cc-pVTZ calculations were performed), and it was found that the whole surface, characterised by the electron density of 0.001 au, possessed a negative electrostatic potential; the EP maxima occurred for the F centres, but they were still negative [49]. Hence, if the F⋯σ contacts observed for the crystal structures presented here (Table 1) are stabilising, this could likely be due to dispersion forces.

**Figure 2:**
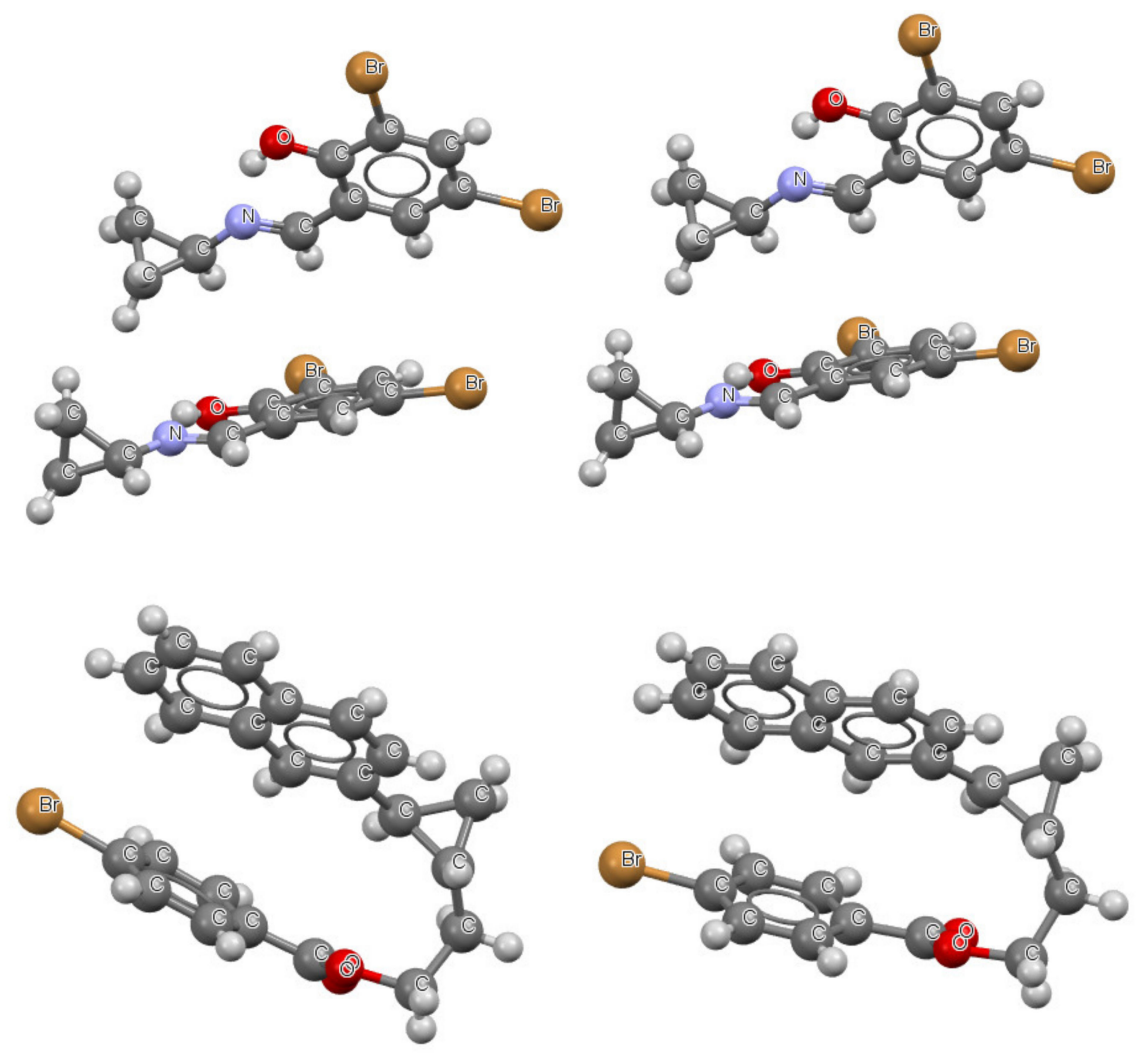
The fragments of the crystal structures of 2,4-dibromo-6-(cyclopropyliminomethyl) phenol (KIKLIA refcode, **upper**) and 2-(2-(2-naphthyl)cyclopropyl)ethyl 4-bromobenzoate (SUTVOU refcode, **below**); small grey circles correspond to H-atoms.

The second search concerned complexes of species containing a cyclopropane ring. Ten crystal structures fulfilling the above-mentioned criteria were found. In five structures, F⋯σ (C–C) interactions were found; in four structures, there were Br⋯σ (C–C) contacts; and in one case, a Cl⋯σ (C–C) interaction was observed. F⋯σ (C–C midpoint) distances in a range from 2.94 to 3.04 Å were observed, the 3.33–3.43 Å range for Br⋯σ distances occurred, and the Cl⋯σ distance was equal to 3.26 Å. One can see that, for the cyclopropane-ring complexes and the dihydrogen cases, the α-angle was very close to 90° for all structures. The β-angle was closer to 180° in the former complexes than in the dihydrogen complexes. Particularly, it was observed for the Br⋯σ interactions that, in extreme cases, this angle was equal to 160° and 166° for structures marked by KIKLIA and SUTVOU refcodes, respectively. This is the experimental evidence for the existence of A–X⋯σ halogen bonds. Figure 2 presents these crystal structures where the Br⋯σ (C–C) contacts occur: 2,4-dibromo-6-(cyclopropyliminomethyl) phenol (KIKLIA refcode) and 2-(2-(2-naphthyl) cyclopropyl) ethyl 4-bromobenzoate (SUTVOU refcode).

**Figure 3:**
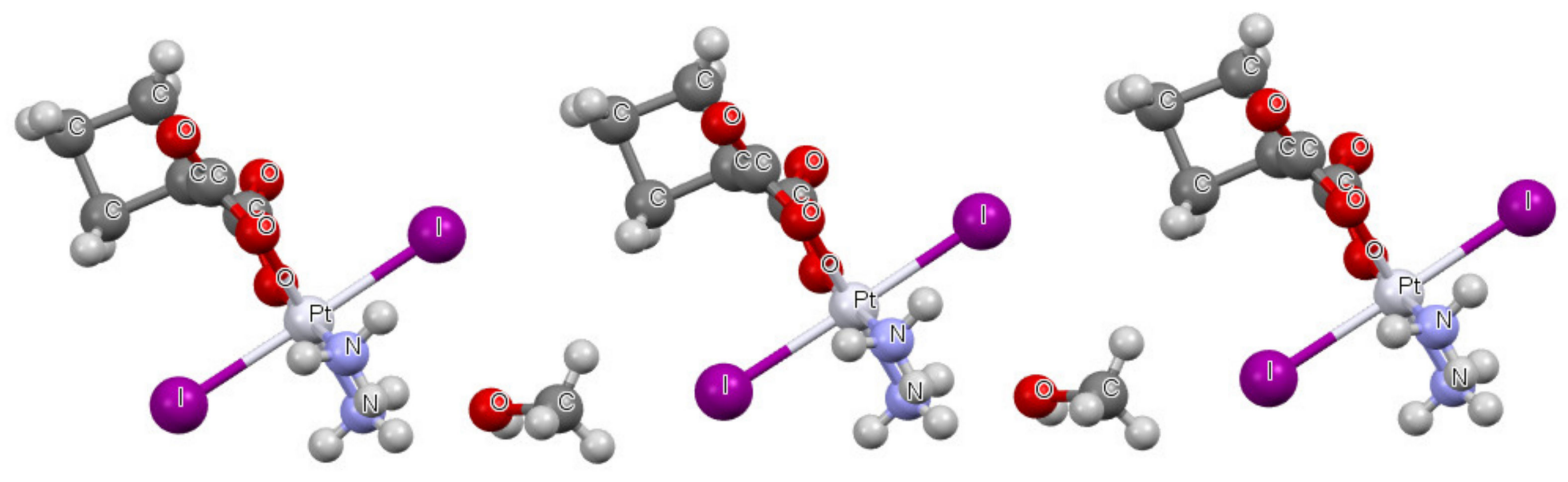
The fragment of the crystal structure of diammine-(cyclobutane-1,1-dicarboxylato)-di-iodo-platinum(iv) methanol solvate (QASKAX refcode); small grey circles correspond to H-atoms.

The next search concerned complexes of the Lewis base units containing the cyclobutane ring. Thirteen crystal structures were found. Only in one case were the I⋯σ (C–C) contacts detected, with the corresponding distance equal to 3.55 Å. The fragment of this structure, diammine-(cyclobutane-1,1-dicarboxylato)-di-iodo-platinum(iv) methanol solvate (QASKAX refcode), is presented in Figure 3. Here, the Pt-I bond is in contact with the C–C bond of the cyclobutane ring. This almost creates the ideal T-shaped structure because the α and β angles are equal to 89.1° and 179.4°, respectively. In another structure, the Cl⋯σ (C–C) contacts were observed with a distance of 3.27 Å; the α and β angles were equal to 89.1° and 165.1°, respectively. For all remaining structures of this search, the F⋯σ (C–C) interactions existed with the corresponding distances in the 2.97–3.05 Å range. The α angles were close to 90° but the β angles were quite far from 180°.

**Figure 4:**
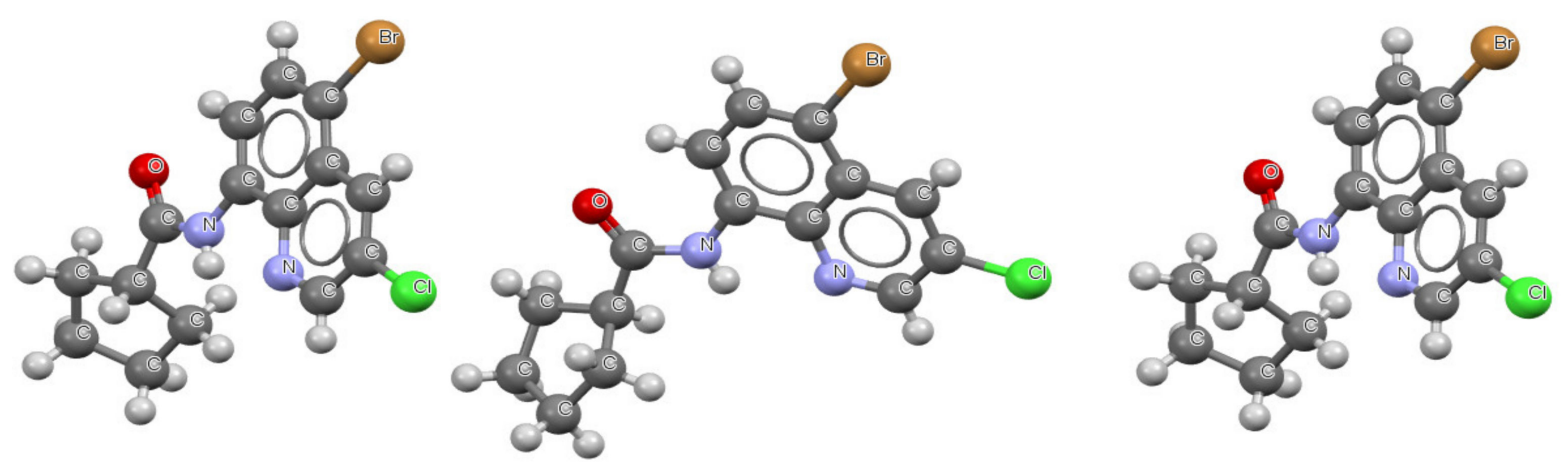
The fragment of the crystal structure of N–(5-bromo-3-chloroquinolin-8-yl) cyclopentanecarboxamide (NARBEX refcode); small grey circles correspond to H-atoms.

In the case of structures with the cyclopentane ring playing the role of the electron donor, only four crystal structures were found. In one structure, Cl⋯σ (C–C) interaction occurred, with Cl⋯σ distances equal to 3.36 Å. This is similar to the desired T-shaped structure because the α and β angles were equal to 89.9° and 170.9°, respectively. The fragment of this crystal structure, N-(5-bromo-3-chloroquinolin-8-yl) cyclopentanecarboxamide (NARBEX refcode), is presented in Figure 4. In the three remaining structures, F⋯σ (C–C) interactions were observed with F⋯σ distances from 3.02 to 3.03 Å. The α angle for these systems were close to 90°, but the β angles were approximately equal to 150°.

The results of searches showed that the greatest representation of X⋯σ interactions concerned F⋯σ interactions, where one could expect contacts between negatively charged sites. This may have indicated that these are rather weak interactions with the dominant dispersion forces; in addition, the β angles for these systems are most often far from 180°. This explains why it is difficult to classify the interactions in these complexes as F⋯σ halogen bonds. However, for several other systems where the heavier halogen atom is in contact with the σ-electrons of the C–C bond, the approximately T-shaped structures are observed because the α and β angles in these systems are close to 90° and 180°, respectively. It proves that these species are linked by A–X⋯σ halogen bonds.

### 2.2. Energies and Geometries of Complexes Linked by A–X⋯σ Interactions

The complexes of the XCCH and X_2_ species (X = F, Cl, Br and I) with molecular hydrogen, cyclopropane, cyclobutane and cyclopentane are analysed theoretically here. Full optimisations were performed for these complexes at the ωB97XD/aug-cc-pVTZ level, and only slight modifications were made to include relativistic effects concerning systems containing iodine. The frequency analysis shows that these systems correspond to energetic minima. For such geometries of optimised complexes, additional CCSD(T)/aug-cc-pVTZ calculations were performed to evaluate the interaction energies at the higher level. The details of the methods applied for all calculations are presented further in Section 3, Computational Approaches.

**Figure 5:**
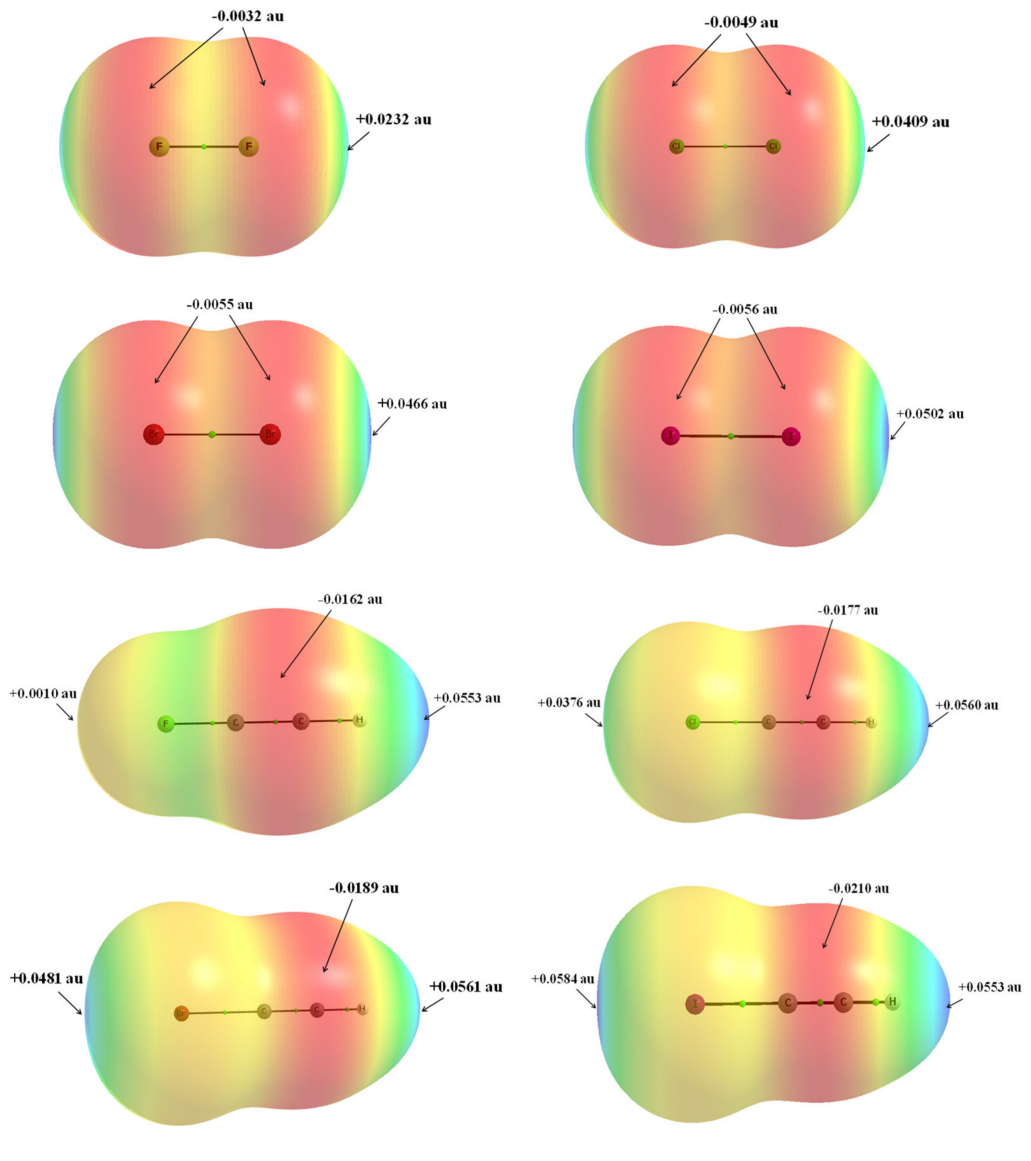
Molecular graphs of X_2_ and HCCX molecules: small green circles correspond to the bond critical points; the EP molecular surfaces characterised by the 0.001 au electron density are presented; blue colour corresponds to the maximum positive EP; red colour corresponds to the negative minimum EP; and the scale of colours concerns each of the molecules separately. According to left-to-right and top-to-bottom order, the following molecules are presented: F_2_, Cl_2_, Br_2_, I_2_, FCCH, ClCCH, BrCCH and ICCH.

The XCCH and X_2_ units may act as Lewis acids through the σ-holes located at the X centre in the extension of the X–X or C–X bond. Figure 5 presents the molecular graphs of the Lewis acid units with the corresponding electrostatic potentials (EPs) mapped on molecular surfaces, characterised by the 0.001 au electron density. For these molecules, the minimum negative EPs occur in the belts of halogen centres around X–X or C–X bonds, whereas the maximum EPs are observed at the above-mentioned σ-holes for the X_2_ molecules; for the HCCX molecules, the EP maxima occur for these σ-holes and for H centres. For the X_2_ molecules, the positive EP value increases with the increase in the atomic number of the halogen, in agreement with earlier studies [5,7]. A similar trend is observed for the HCCX units; the increase in EP values for the σ-hole for heavier halogen centres. The positive EP for the X centre in the FCCH species is negligible, +0.001 au, but for iodine in the ICCH unit it amounts to +0.058 au (the total EP maximum), more than EP at the hydrogen centre (+0.055 au). However, the total EP maxima are observed at the H centre for the FCCH, ClCCH and BeCCH species, whereas the local EP maxima occur for the halogen centres.

**Figure 6:**
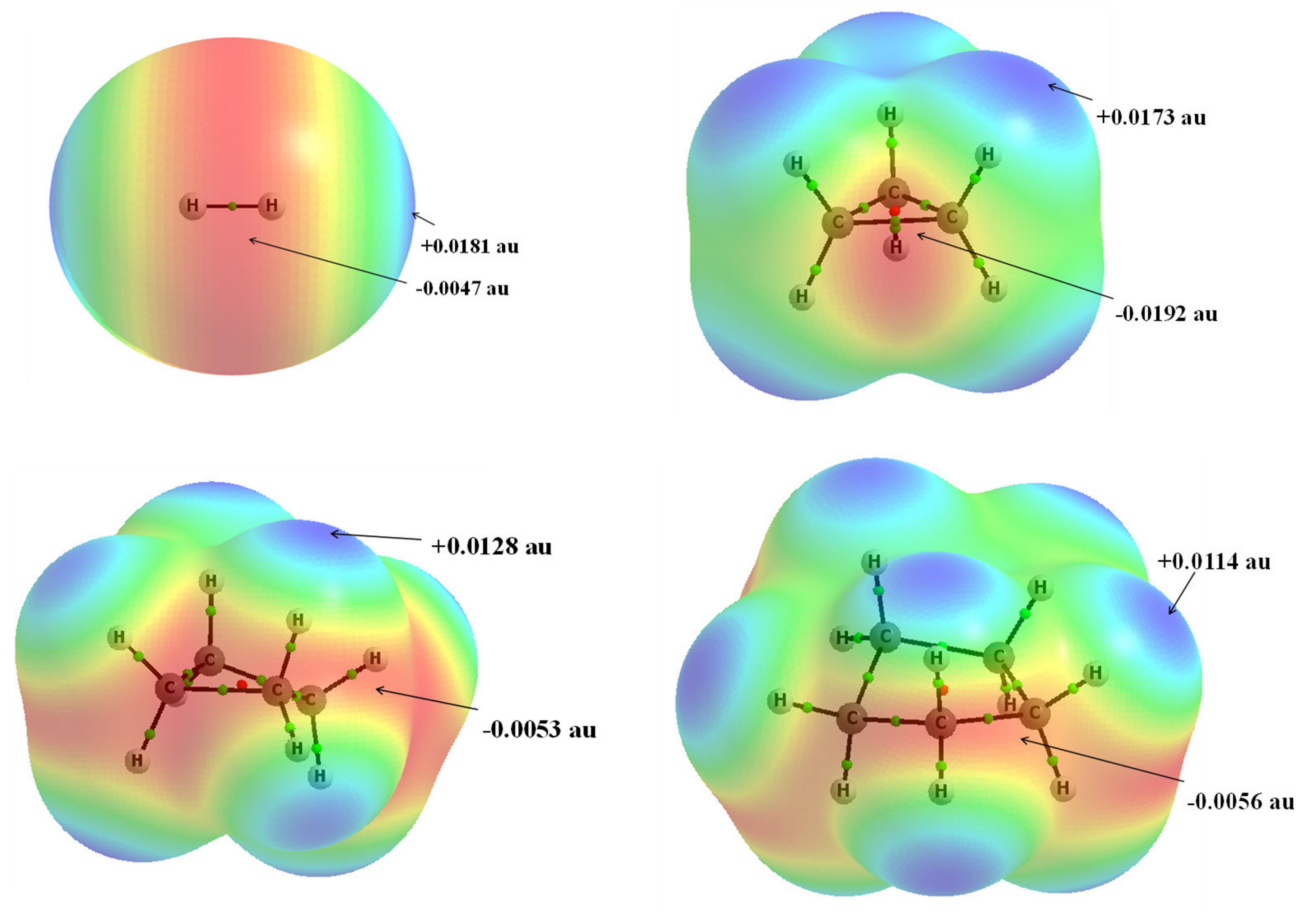
Molecular graphs of H_2_, C_3_H_6_, C_4_H_8_ and C_5_H_10_ molecules: small green circles correspond to the bond critical points; the EP surfaces characterised by the 0.001 au electron density are presented; blue colour corresponds to the maximum positive EP; red colour corresponds to the negative minimum EP; and the scale of colours concerns each of the molecules separately. According to left-to-right and top-to-bottom order, the following molecules are presented: H_2_, C_3_H_6_, C_4_H_8_ and C_5_H_10_.

Figure 6 shows the molecular graphs of units that act as the electron donors through σ-bonds (H–H or C–C bonds). These are the following species: dihydrogen, cyclopropane, cyclobutane, and cyclopentane.

Figure 6 shows that for all species which can act as the Lewis bases, the hydrogen atoms are characterised by the maximum positive EP values, whereas the minima are attributed to the σ-bonds: H–H and C–C. The greatest absolute value corresponding to the minimum EP occurs for cyclopropane. In the latter structure, the C–C–C angles are equal to 60°, which means that the electron charge is not concentrated along the line connecting the nuclei, but it is accumulated outside the C–C links [50]. This results in stronger Lewis base properties of the latter species, in comparison with the dihydrogen and other cycloalkanes. In general, the analysis of the EP surfaces for the potential Lewis acid–base units shows that the halogen centres may act as Lewis acids through σ-holes, whereas H–H and C–C bonds may act as the Lewis base centres. In the case of the HCCX species, the H centres can interact with Lewis bases instead of the halogens, forming hydrogen bonds. However, such A–H⋯σ hydrogen bonds were not a subject of this study; these interactions have been analysed before [33,34,35].

**Figure 7:**
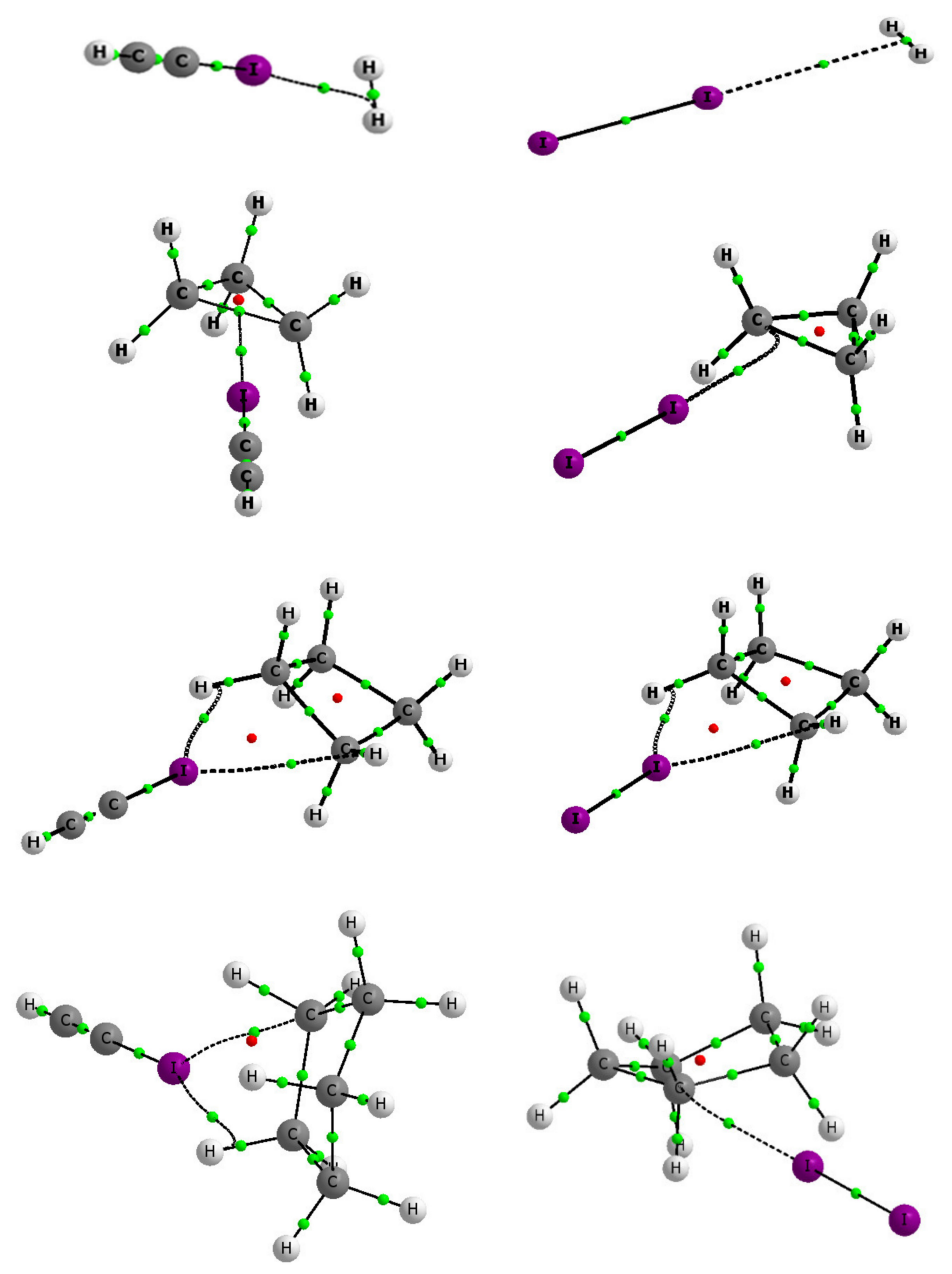
The molecular graphs of the complexes of ICCH and I_2_: big circles correspond to attractors; small green circles to bond critical points; small red circles to ring critical points; and solid and broken lines to bond paths. According to left-to-right and top-to-bottom order, the following molecules are presented: HCCI⋯H_2_, I_2_⋯H_2_, HCCI⋯C_3_H_6_, I_2_⋯C_3_H_6_, HCCI⋯C_4_H_8_, I_2_⋯C_4_H_8_, HCCI⋯C_5_H_10_ and I_2_⋯C_5_H_10_.

The molecular graphs of complexes between halogen systems and dihydrogen or cyclopropane systems confirm the expectations of the Lewis acid–base properties of the units previously discussed. Figure 7 shows the molecular graphs of the ICCH and I_2_ complexes. The species where other halogen centres play a role in the electron acceptor are characterised by similar molecular graphs. The Quantum Theory of ´Atoms in Molecules´, (QTAIM) characteristics are discussed further here. One can see that interactions which occur in complexes may be classified as the A–X⋯σ halogen bonds, because the halogen centre is directed to the σ-bonds, H–H or C–C (Figure 7). In two cases of the complexes of C_5_H_10_-F_2_ and C_5_H_10_-FCCH, the optimisations led to configurations where halogen bonds did not occur. The interactions that may be classified as hydrogen bonds occurred in these systems. These complexes were not analysed here.

Table 2 presents the interaction energies [51] (marked as E_int_^1^’s and E_int_^2^’s, for ωB97XD/aug-cc-pVTZ and CCSD(T)/aug-cc-pVTZ//ωB97XD/aug-cc-pVTZ results, respectively—see Section 3 for details of methods of calculations) corrected for the basis set superposition error, BSSE [52]. The |E_int_^1^| and |E_int_^2^| values do not exceed 3 kcal/mol, which means that the A–X⋯σ halogen bonds are rather weak interactions. A similar situation occurs for the A–H⋯σ hydrogen bonds [35] that are usually weaker than the A–H⋯π and A–X⋯B interactions. One can see that, among the halogen-bonded systems discussed, the interactions occurring in the C_3_H_6_-I_2_ and C_3_H_6_-ICCH complexes are the strongest, because the corresponding |E_int_^1^| and |E_int_^2^| values are close to 3 kcal/mol.

Table 2 shows that the CCSD(T) interaction energies, E_int_^2^’s, are slightly “more negative” than the ωB97XD energies, E_int_^1^’s. However, there is an excellent correlation between them because the linear correlation coefficient R is equal to 0.97. The interactions in complexes of cyclopropane are stronger than the corresponding interactions in complexes of other Lewis base units. A similar situation occurs for the corresponding hydrogen-bonded systems, where the strongest interactions with cyclopropane are observed [35,50,53]. The relatively strong interactions occur for hydrogen-bonded complexes of cyclopropane and tetrahedrane because of their unusual properties; they possess few characteristics of unsaturated hydrocarbons [50,53]. It was mentioned before that in the structure of cyclopropane, the C–C–C angles are equal to 60°, which means that the electron charge is not concentrated along the line connecting the nuclei, but it is accumulated outside the C–C links [50].

In the systems analysed here, the weakest halogen bonds are observed for the complexes of dihydrogen; the |E_int_| values do not exceed 1 kcal/mol for both methods of calculations applied. In the complexes of cyclobutane, the |E_int_| values are usually situated between 1 kcal/mol and 2 kcal/mol; in two cases of the C_4_H_8_-F_2_ and C_4_H_8_-FCCH systems, these values are lower than 1 kcal/mol. Slightly stronger interactions than those in the complexes of cyclobutane occur for species containing the cyclopentane unit. For the C_5_H_10_-I_2_ and C_5_H_10_-ICCH complexes, the |E_int_^1^| and |E_int_^2^| values are greater than 2 kcal/mol. The basis set superposition error corrections do not exceed 0.05 kcal/mol for complexes of dihydrogen and 0.15 kcal/mol for the remaining halogen-bonded systems if the ωB97XD/aug-cc-pVTZ calculations are considered. The greater BSSE values are observed for the CCSD(T) results; they sometimes exceed 2 kcal/mol (see Table S2 in Supplementary Information).

Table 2 presents the distances between the X halogen centre and the σ-bond of the Lewis base (the midpoint of the H–H or C–C bond), X⋯σ; distances between 3.1 Å and 3.65 Å are observed. The shortest distances occur for the H_2_–X_2_ complexes, between 3.11 Å and 3.24 Å. However, for the C_3_H_6_–Cl_2_ complex, a short distance equal to 3.19 Å is observed. It is worth noting that various halogen centres occur in the complexes analysed here; they are characterised by different van der Waals radii. The σ-bonds that act as electron donors also possess different properties. This is the reason why it is difficult to compare the X⋯σ distances.

Table 2 shows the α and β angles (see Scheme 3) for complexes analysed theoretically; these angles indicate the approximate T-shaped structures. These angles are equal or close to 90° and 180°, respectively, particularly for the systems of dihydrogen and cyclopropane. In the case of dihydrogen, the electron acceptors have free access to the H–H electron-donating σ-bond, whereas for cyclopropane, the electron density accumulation occurs outside of the C–C bonds which results in greater negative EP values for the corresponding bonds´ regions. In the case of the cyclobutane and cyclopentane complexes, ‘less negative’ EP values are observed at C–C bonds than for cyclopropane. These results clearly show that the bonds containing halogen centres are directed towards the H–H and C–C σ-bonds, which confirms the occurrence of the A–X⋯σ halogen bonds. A slightly different situation occurs in the crystal structures discussed earlier. For the A–X⋯σ arrangements in crystals, the β angles are often far from 180°. Particularly, such non-linearity is observed for B-F⋯σ contacts of the BF_4_^−^ anions. In addition, the crystal packing may disturb the geometries of the A–X⋯σ arrangements. Despite these factors, the T-shaped geometries confirming the existence of the A–X⋯σ interactions are observed even in crystal structures, especially for heavier halogen centres.

Table S2 contains the C–X and X–X bond stretching frequencies and the corresponding intensities. These bonds participate directly in the formation of halogen bonds with the σ-bond electrons of the Lewis base units. It has often been claimed in numerous studies that, in the case of the A–H⋯B hydrogen bond, the most common effect resulting from its formation is the elongation of the proton-donating A–H bond and, consequently, the shift of the corresponding stretching frequency to a lower value (red shift) [54,55]. However, the shortening of this bond is sometimes observed as a result of a hydrogen bond formation with the corresponding blue shift [56,57,58,59,60]. The latter effect is rather rare compared to A–H bond elongation. Similarly, both cases of elongation and the shortening of the A–X bond in A–X⋯B halogen bonds have been described in various studies [61,62]; however, the blue shift occurs here more frequently than in hydrogen bonds.

In the case of the complexes analysed in this study, the elongation of the A–X bonds (C–X or X–X) was observed. They were shortened only in few cases; however, this reduction in length was insignificant (see Table S2). The changes in the A–X bond lengths are not pronounced significantly (they do not exceed 0.3%), and they are accompanied by the corresponding changes in stretching frequencies that are similarly very small (not exceeding 2%). The blue shifts in halogen bonds analysed in this study are observed only in the case of the FCCH complexes where fluorine acts as the Lewis acid centre.

### 2.3. C–X⋯σ and X–X⋯σ Interactions in Complexes of Dihydrogen and Cyclopropane

The Lewis acid–Lewis base interactions lead to electron charge shifts from the base unit to the acid unit; a rearrangement of the electron density within these units is also observed [22]. These shifts and rearrangements are greater for stronger interactions, and they testify to their covalent character. Such shifts are related to the charge transfer and polarisation interaction energy terms, or to the orbital–orbital interactions within the Natural Bond Orbital (NBO) approach [63,64]. However, these shifts and related orbital–orbital energies are close to zero for the cyclobutane and cyclopentane complexes. For the remaining species analysed in this study, the complexes of dihydrogen and cyclopropane, the electron charge shifts are more pronounced but remain small and often negligible. This explains why only for the dihydrogen and cyclopropane complexes are the charge shifts discussed in this section.

Table 3 presents selected NBO [63,64] and Quantum Theory of Atoms in Molecules (QTAIM) [65,66] parameters for the complexes of dihydrogen and cyclopropane. For example, charges of the Lewis base units in complexes are collected here. Almost in all cases of the complexes, these charges are equal to or very close to zero, meaning that the electron charge transfer as an effect of complexation does not occur or is negligible. The most important transfers, from the Lewis base unit to the Lewis acid unit, occur for the C_3_H_6_-Br_2_ and C_3_H_6_-I_2_ complexes, and remain small; about 30 and 20 millielectrons, respectively (Table 3).

The electron charge transfers are accompanied by the orbital–orbital interactions. It is well known that in the case of the A–H⋯B hydrogen bond, the n(B)→σ_AH_ * overlap is most important, often treated as a signature for the existence of this kind of interaction [63,64]. The n(B) marks the lone electron pair orbital of the B centre that plays a role of the proton acceptor, while σ_AH_* is the antibonding orbital of the A–H proton-donating bond. In the case of the A–H⋯σ kind of hydrogen bond, the σ→σ_AH_* overlap is observed where σ corresponds to the bond of the Lewis base unit that acts as the donor of electron charge [33,35]. The similar σ_HH/CC_→σ_AX_ * overlap occurs here for the A–X⋯σ halogen bonds (A–X designates, X-X, or C–X bond). The σ_HH/CC_ orbital corresponds to the H–H or C–C bond of dihydrogen or cycloalkane. However, the corresponding orbital–orbital interaction energies are low and do not exceed 0.6 kcal/mol (Table 3). The strongest orbital–orbital interactions occur for the H_2_-ICCH and H_2_-Br_2_ complexes where energies amount to approximately 0.6 kcal/mol; they correspond to σ_HH_→σ_CI_ * and σ_HH_→σ_Br2_ * overlaps, respectively. There are other overlaps aside from those mentioned above; however, additional overlaps occur only for the C_3_H_6_-ICCH, C_3_H_6_-Cl_2_, C_3_H_6_-Br_2_, and C_3_H_6_-I_2_ complexes (Table S3 in Supplementary Information).

Table 3 presents the electron densities at the bond critical points (BCPs) corresponding to the X⋯σ bond path. Such bond paths link the halogen centre attractor (X) with the BCP of the H–H or C–C bond of dihydrogen or cyclopropane, respectively. However, in the few cases of the complexes of H_2_ and C_3_H_6_, the bond path that links Lewis acid and Lewis base units is not connected with the BCP of the H–H or C–C bond, but turns to one of the attractors, hydrogen or carbon, respectively. Figure 7 presents this situation for the H_2_-ICCH and C_3_H_6_-I_2_ complexes. These cases are treated as similar to those where bond paths correspond to attractor–BCP links. The situation is much more complicated for complexes of cyclobutane and cyclopentane (see Figure 7) where the straight bond paths linking the X centre of Lewis acid with the BCP of the C–C bond of Lewis base are not observed; instead, other types of bond paths occur. It is worth mentioning that the significance and the physical meaning of the bond path have been the subjects of numerous discussions and polemics [67,68,69,70,71]. It was mentioned that bond paths correspond to the local stabilising interactions [70,71]. This may explain why numerous links (bond paths) occur for the weak interactions between Lewis acid–base units.

The electron density of BCP, ρ_BCP_, corresponding to the X⋯σ bond path (X⋯BCP or X⋯attractor bond path) is rather low (Table 3). The greatest ρ_BCP_ values of 0.008 au are observed for the C_3_H_6_-Br_2_ and C_3_H_6_-I_2_ complexes. It is known that the ρ_BCP_ value often correlates with other parameters corresponding to the strength of interaction [72,73,74]. For the water trans-liner dimer, the electron density of the H⋯O BCP corresponding to the intermolecular link amounts to 0.023 au, whereas the interaction energy is equal to −4.5 kcal/mol (MP2/6-311++G(d,p) level of calculations) [75]. The low values of ρ_BCP_ for halogen-bonded complexes indicate that these interactions are weak. The latter is in agreement with the energetic results because the absolute values of interaction energies do not exceed 3 kcal/mol (Table 2). One can see that, for the cyclobutane and cyclopentane complexes, connections between the Lewis acid and Lewis base units are ambiguous (Figure 7) because they are not single bond paths, as is the case for the dihydrogen and cyclopropane complexes.

In summary, the complexes of dihydrogen and cyclopropane were discussed in this section because the most important electron charge shifts resulting from complexation occur in these species. However, these shifts remain small, as is the case for the other parameters related to the charge shifts. The most important orbital–orbital interactions, as well as the electron densities at the BCPs, which correspond to the intermolecular links, also remain small. The values of the above parameters are related to the covalent characteristics of interactions [22]. This explains why the additional approach, energy decomposition analysis, EDA [76,77], was used here—to deepen the understanding of the characteristics of A–X⋯σ interactions.

### 2.4. Energy Decomposition Analysis

Energy decomposition analysis, EDA [76,77], was performed for the selected complexes discussed here. The selection criterion was based on the interaction energy, E_int_ (Table 2). This decomposition was carried out for those systems where |E_int_^1^| ≥ 1.5 kcal/mol and |E_int_^2^| ≥ 1.8 kcal/mol. For weaker interactions, the energy partitioning could lead to terms of energies close to the accuracy of their determination. The results of the decomposition for five complexes of cyclopropane, three complexes of cyclobutane, and three complexes of cyclopentane are presented in Table 4. In the case of the cyclopropane complexes, especially with molecular halogens, i.e., for C_3_H_6_-Cl_2_, C_3_H_6_-Br_2_, and C_3_H_6_-I_2_, the approximate equivalency of attractive interaction energy terms is observed. In two cases, the C_3_H_6_-BrCCH and C_3_H_6_-ICCH complexes, the dispersion term, ΔE_disp_, is the most important attractive term; followed by the electrostatic term, ΔE_elstat_; and finally, the least important term is the energy related to the electron charge shifts, ΔE_orb_. In the case of the six remaining complexes of cyclobutane and cyclopentane, the dispersion interaction is also the most important attractive contribution, amounting to ~50%. For these six complexes, the following order of the importance of the interaction energies can be presented: |ΔE_disp_| > |ΔE_orb_| > |ΔE_elstat_|.

One can see that the interaction energies, ΔE_int_, presented in Table 4, differ from those collected in Table 2 (E_int_^1^ and E_int_^2^). However, the EDA calculations were performed at different levels (see Section 3, Computational Approaches for details) from the results presented so far. In spite of the different levels applied, there is a linear correlation between ΔE_int_ and E_int_^1^ values (R = 0.97). This means that the relationships between the strengths of the interactions of complexes presented in Table 4 are preserved, and the discussion on interaction energy contributions is in line with other results discussed so far in this study.

It was pointed out that the importance of all interaction energy terms increases with the increase in the strength of interaction, which is related to the total interaction energy [19,20,22]. However, such an increase is not uniform. For example, it was found that for the A–H⋯B hydrogen bond, the shortening of the H⋯B distance connected with the increase in the strength of the interaction is related to the increase in the importance of electrostatic and delocalisation energies [78,79]. However, the delocalisation interaction energy which expresses the electron charge shifts (similarly to the ΔE_orb_ term in the decomposition applied here) increases more than the electrostatic energy, with the shortening of this distance. In recent studies, it was found that the Pauli repulsion term correlates with the sum of all attractive terms; this is observed for hydrogen, tetrel, and triel bonds [22].

Similar tendencies occurred for the A–X⋯σ halogen bonds analysed in this study. Figure S2 (Supplementary Information) shows the linear correlation between the repulsion term, ΔE_Pauli_, and the sum of the attractive terms: ΔE_elstat_ + ΔE_orb_ + ΔE_disp_. This correlation is observed in spite of the different halogen Lewis acid centres in the sample of complexes analysed. In addition, the separated orbital energy term, ΔE_orb_, well correlates with the repulsion term, ΔE_Pauli_, whereas the linear correlation between the electrostatic term, ΔE_elstat_, and the repulsion is not good, and the dispersion attraction does not correlate with the repulsion, although for almost all complexes the dispersion term is the most important attractive term.

Table 4 shows that the orbital energy which expresses electron charge shifts is only one of the attractive components, and not the most important term. This explains why the charges, overlap energies, and electron densities at the BCPs, related to the charge transfers from the Lewis base to the Lewis acid presented in the former section, are so small.

## 3. Computational Approaches

The calculations were carried out with the Gaussian16 set of codes [80]; the ωB97XD functional [81,82] and the aug-cc-pVTZ basis set [83] were applied. It has been justified that the ωB97XD functional provides more reliable results than other functionals which are commonly used [82], especially in connection with the aug-cc-pVTZ basis set. The calculations for iodine were completed with quasi-relativistic small-core effective core potentials (ECPs) [84] and the corresponding Peterson AVTZ basis set [85]. Frequency calculations were performed at the same level as geometry optimisations; the optimised structures corresponded to the energetic minima, because no imaginary frequencies were found. The Counterpoise Correction approach was used to estimate the basis set superposition error, BSSE [52], for complexes discussed in this study. The interaction energy was calculated as the difference between the energy of the complex and the sum of the energies of monomers in the complex [51] (E_int_^1^ values—Table 2).

For the above-mentioned ωB97XD/aug-c-pVTZ geometries of complexes that corresponded to energetic minima, the CCSD(T) method [86] was applied because single-point CCSD(T)/aug-cc-pVTZ calculations were performed. The latter calculations were used to evaluate the interaction energies (E_int_^2^ values—Table 2).

The ‘Quantum Theory of Atoms in Molecules’, QTAIM [65,66], calculations were also performed to analyse bond paths related to the intermolecular interactions and corresponding bond critical points. The AIMAll [87] program was used to carry out QTAIM calculations on the ωB97XD/aug-c-pVTZ wave functions corresponding to the optimised structures.

The BP86-D3/TZ2P level was applied to perform the energy decomposition analysis (EDA) calculations. For this, the BP86 functional [88,89] with the Grimme dispersion corrections [90] and the uncontracted Slater-type orbitals (STOs) as basis functions with triple-ζ quality for all elements [91] were used. These BP86-D3/TZ2P level results of calculations are in agreement with the ωB97XD/aug-c-pVTZ results; particularly with regard to the interaction energies (see Section 2, Results). The BP86-D3/TZ2P level was chosen for EDA calculations because it was checked and the results corresponded to other DFT calculations; additionally, convergence of the energy terms is often achieved here [35]. The decomposition calculations [76,77] were carried out with the use of ADF2019.302 program codes [92]. The total interaction energy was decomposed here into terms according to Equation (1) given below:

ΔE_int_ = ΔE_elstat_ + ΔE_Pauli_ + ΔE_orb_ + ΔE_disp_ (1)

The ΔE_elstat_ attractive term corresponds to the electrostatic interaction between the unperturbed charge distributions of atoms. The Pauli repulsion, ΔE_Pauli_, is the energy change related to the transformation from the superposition of the unperturbed electron densities of the isolated fragments to the wave function. This properly obeys the Pauli principle through the antisymmetrisation and renormalisation of the product wave function. The orbital interaction, ΔE_orb_, corresponds to the charge transfer and polarisation, i.e., to electron charge shifts that result from the complexation. The dispersion interaction energy term, ΔE_disp_, is also included (Equation (1)). The attractive energy terms are negative, whereas the repulsive terms (the Pauli repulsion) are positive.

The NBO method [63,64] was used to calculate the atomic charges and energies of orbital–orbital interactions. The NBO 6.0 program [93] implemented in the ADF2019 set of codes [92] was applied to perform NBO calculations. The NBO calculations and the energy decomposition analysis were carried out at the BP86-D3/TZ2P level for geometries optimised at the ωB97XD/aug-cc-pVTZ level. For iodine elements that occurred in some of species analysed, the ZORA approach [77] was applied to include relativistic effects. The BP86-D3/TZ2P level was applied for the EDA and NBO calculations because it was partly forced by the corresponding subroutines that were inserted in the ADF program; this program applies Slater-type basis sets, whereas the Dunning-style basis set was applied in optimisation with the Gaussian 16 program. However, the consistency of the BP86-D3/TZ2P and ωB97XD/aug-cc-pVTZ levels was previously justified in this study.

## 4. Conclusions

The A–X⋯σ halogen bonds that were characterised by the σ-bonds, H–H or C–C, acting as the Lewis base sites, were analysed here. The molecular hydrogen and cycloalkanes were chosen as the Lewis base units. It was found that the complexes of cyclopropane are characterised by the strongest halogen bonds in comparison with complexes of dihydrogen, cyclobutane and cyclopentane. The analyses of the energies of interactions, as well as of the bond paths linking monomers in the complexes, showed that the systems of dihydrogen and cyclopropane may be classified as the halogen-bonded complexes, but the systems of cyclobutane and cyclopentane are debatable due to the nature of their interactions. For the latter complexes, the dispersion forces are dominant attractive interactions, whereas in complexes of cyclopropane and dihydrogen, other attractive interactions, both orbital and electrostatic, are also very important. The results of the decomposition of the energies of the interactions showed that the Pauli repulsion correlates well with the attractive orbital energy; a worse correlation with the electrostatic energy was observed and there was no correlation between repulsion energy and dispersion energy. The orbital energy related to the electron charge shifts is only one of the attractive components, and not the most important term. This explains why the charge shifts, overlap energies and electron densities at BCPs, which reveal the covalent characteristics of interactions, are so small.

The other important findings of this study concern the experimental evidence of the existence of A–X⋯σ halogen bonds. These links observed in numerous crystal structures were revealed by the results of CSD searches. However, surprisingly, the main part of X⋯σ halogen bonds is related to the A–F⋯σ arrangements, where two sites in contact with each other are negatively charged. Table 2 shows that the interaction energies for the complexes characterised by the F⋯σ bond, |E_int_|, do not exceed 0.9 kcal/mol (CCSD(T) results). The contacts between the two negatively charged sites, as well as the negligible electron transfer (if any) in these complexes suggest that they are linked by dispersion forces. It is worth mentioning that the greatest electron transfer for complexes with the fluorine Lewis acid centre occurs for the C_3_H_6_-F_2_ complex and amounts to only 0.01 au. It has been justified that EDA for fluorine complexes was not performed because they are linked by weak interactions. However, the remaining results indicate that the A–F⋯σ arrangements are stabilised by dispersion forces.

The A–X⋯σ arrangements in crystal structures which concern heavier halogen atoms, chlorine, bromine and iodine, exhibit T-shaped structures in several cases where the A–X bond is directed to the midpoint of the single C–C bond. This may be treated as experimental evidence for A–X⋯σ halogen bonds.

## Figures and Tables

**Table 1:** The crystal structures that contain A–X···σ halogen bonds: refcodes, e.s.ds values (Å), types of A–X bonds, X···σ distances (Å), and α and β angles (in degrees, see Scheme 3) are given.

Refcode	Max e.s.d.	A–X	X···σ	α	β
Dihydrogen as an electron donor					
NOLJIH	0.01	B-F	2.432	86.4	113.7
NOLJIH01	0.01	B-F	2.539	83.8	125.7
NOLJIH02	0.01	B-F	2.432	86.4	113.6
XAXMIT	0.01	B-F	2.467	87.6	111.6
Cyclopropane ring as an electron donor					
HEDQAL	0.005	C-F	2.941	85.3	149.5
HEDQIT	0.005	C-F	2.940	85.3	149.5
HIGNAO02	0.03	Al-Br	3.410	87.5	145.5
KIKLIA	0.01	C-Br	3.332	89.6	159.7
OMIREI	0.03	C-Br	3.430	89.4	157.1
PILSEK	0.005	C-F	3.038	89.9	127.1
QUSREC	0.01	C-F	3.038	89.9	148.6
RUNQEW	0.005	C–Cl	3.262	88.7	150.1
SUTVOU	0.005	C-Br	3.372	89.2	165.9
UFIYEP	0.005	C-F	2.982	87.0	126.4
Cyclobutane ring as an electron donor					
DEZDUH	0.01	C-F	3.025	89.9	133.5
EQIXAF	0.03	P-F	3.012	89.9	123.8
EQIXIN	0.03	P-F	2.989	89.9	125.5
GEGDUU	0.01	C-F	3.054	89.6	159.7
GOGXOP	0.005	C-F	2.993	88.4	147.7
HEXYIV	0.005	C-F	2.971	89.4	122.7
PUFSOA	0.005	C-F	3.011	89.6	155.8
QASKAX	0.03	Pt-I	3.551	89.1	189.4
QUBGOJ	0.01	B-F	3.012	90.0	165.4
SITZOK	0.005	C-F	3.022	89.8	153.5
TOHDAY	0.005	C–Cl	3.265	89.1	165.1
TORPOX	0.005	C-F	3.004	89.1	146.5
YAMGAU01	0.005	C-F	3.001	88.6	151.0
Cyclopentane ring as an electron donor					
XACHOC	0.005	C-F	3.020	89.3	153.2
HAFQEN	0.005	C-F	3.024	89.6	149.4
NARBEQ	0.005	C–Cl	3.364	89.9	170.9
VANNUT	0.01	B-F	3.036	90.0	148.0

**Table 2:** The characteristics of interactions in complexes analysed: interaction energy corrected for BSSE (in kcal/mol)—E_int_^1^, and E_int_^2^ are specified; the distance between the halogen centre and the midpoint of the H–H or C–C bond (in Å)—X⋯σ; as well as α and β angles defined in Scheme 3 (in degrees).

Complex	E_int_^1^	E_int_^2^	X···σ	α	β
H_2_-FCCH	−0.09	−0.10	3.482	86.0	179.6
H_2_-ClCCH	−0.23	−0.33	3.474	90.0	180.0
H_2_-BrCCH	−0.38	−0.48	3.295	89.9	179.9
H_2_-ICCH	−0.66	−0.62	3.248	87.2	177.3
H_2_-F_2_	−0.13	−0.18	3.240	89.7	179.6
H_2_-Cl_2_	−0.31	−0.48	3.134	90.0	180.0
H_2_-Br_2_	−0.52	−0.57	3.110	89.9	179.8
H_2_-I_2_	−0.72	−0.60	3.223	89.8	180.0
C_3_H_6_-FCCH	−0.37	−0.48	3.486	90.0	180.0
C_3_H_6_-ClCCH	−1.16	−1.63	3.394	90.0	180.0
C_3_H_6_-BrCCH	−1.86	−2.14	3.377	90.0	180.0
C_3_H_6_-ICCH	−2.79	−2.79	3.445	90.0	180.0
C_3_H_6_-F_2_	−0.57	−0.89	3.165	89.9	179.9
C_3_H_6_-Cl_2_	−1.64	−2.15	3.193	90.0	179.6
C_3_H_6_-Br_2_	−2.36	−2.63	3.241	89.8	179.3
C_3_H_6_-I_2_	−2.97	−2.97	3.399	89.4	179.4
C_4_H_8_-FCCH	−0.45	−0.59	3.455	90.0	179.9
C_4_H_8_-ClCCH	−0.85	−1.30	3.539	90.0	180.0
C_4_H_8_-BrCCH	−1.30	−1.62	3.525	90.0	179.9
C_4_H_8_-ICCH	−1.92	−1.99	3.610	89.9	179.8
C_4_H_8_-F_2_	−0.45	−0.64	3.390	87.2	165.5
C_4_H_8_-Cl_2_	−0.98	−1.53	3.409	88.3	170.9
C_4_H_8_-Br_2_	−1.47	−1.83	3.449	89.9	179.4
C_4_H_8_-I_2_	−1.99	−2.07	3.600	89.9	179.3
C_5_H_10_-ClCCH	−0.89	−1.32	3.571	89.7	179.9
C_5_H_10_-BrCCH	−1.36	−1.64	3.564	87.8	171.4
C_5_H_10_-ICCH	−2.01	−2.03	3.643	86.9	171.0
C_5_H_10_-Cl_2_	−1.04	−1.54	3.470	85.5	164.9
C_5_H_10_-Br_2_	−1.55	−1.85	3.522	84.8	164.9
C_5_H_10_-I_2_	−2.10	−2.13	3.642	85.5	168.0

**Table 3:** The characteristics related to the electron charge transfer: the NBO charge of the Lewis base unit (au), Q_Lb_, the energy of σ_HH/CC_→σ_AX_ *, orbital–orbital interaction (kcal/mol), and the electron density at X⋯σ (or X⋯H/C) BCP (in au), ρ_BCP_.

Complex	Q_Lb_	σ_HH/CC_→σ_AX_ *	ρ_BCP_
H_2_-FCCH	0.00	0	0.001
H_2_-ClCCH	0.00	0.11	0.002
H_2_-BrCCH	0.00	0.29	0.004
H_2_-ICCH	0.00	0.57	0.006
H_2_-F_2_	0.00	0.06	0.001
H_2_-Cl_2_	0.00	0.34	0.004
H_2_-Br_2_	0.01	0.55	0.006
H_2_-I_2_	0.01	0.1	0.006
C_3_H_6_-FCCH	0.00	0	0.002
C_3_H_6_-ClCCH	0.00	0.06	0.005
C_3_H_6_-BrCCH	0.01	0.12	0.006
C_3_H_6_-ICCH	0.01	0.26	0.007
C_3_H_6_-F_2_	0.01	0.07	0.004
C_3_H_6_-Cl_2_	0.02	0.29	0.007
C_3_H_6_-Br_2_	0.03	0.41	0.008
_C_3_H_6_-I_2_	0.02	0.37	0.008

**Table 4:** The total interaction energy and its terms (see Section 3, Computational Approaches) for selected complexes analysed in this study, all expressed in kcal/mol. The percentage contributions of the attractive terms are given (in relation to the sum of all attractive interaction energy terms).

Complex	ΔE_int_	ΔE_Pauli_	ΔE_elstat_	ΔE_orb_	ΔE_disp_	% ΔE_elstat_	% ΔE_orb_	% ΔE_disp_
C_3_H_6_-BrCCH	−2.23	2.97	−1.79	−1.26	−2.16	34	24	41
C_3_H_6_-ICCH	−3.47	4.13	−2.53	−2.23	−2.83	33	29	37
C_3_H_6_-Cl_2_	−2.41	3.48	−2.06	−2.01	−1.83	35	34	31
C_3_H_6_-Br_2_	−3.32	4.34	−2.57	−2.64	−2.45	34	34	32
C_3_H_6_-I_2_	−4.08	4.88	−2.88	−3.10	−2.97	32	35	33
C_4_H_8_-ICCH	−2.60	2.83	−1.17	−1.49	−2.76	22	27	51
C_4_H_8_-Br_2_	−1.93	2.60	−1.06	−1.16	−2.31	23	26	51
C_4_H_8_-I_2_	−2.78	3.01	−1.25	−1.70	−2.84	22	29	49
C_5_H_10_-ICCH	−2.73	2.95	−1.23	−1.58	−2.88	22	28	51
C_5_H_10_-Br_2_	−2.11	2.76	−1.15	−1.37	−2.35	24	28	48
C_5_H_10_-I_2_	−3.00	3.24	−1.36	−1.93	−2.95	22	31	47

## Data Availability

Not applicable.

## Supplementary Materials

The following are available online, Figure S1: The dependence between %Δr and %Δν, the correlation for cyclopropane complexes is shown (black circles), Figure S2: The relationship between the sum of attractive interaction energy terms and the Pauli repulsion term (white circles). Relationships between the Pauli repulsion and separated attractive terms are presented; orbital (white squares), electrostatic (black circles), dispersion (black triangles),Table S1: The crystal structures that contain A-X···σ halogen bonds; refcodes, maximum e.s.d values (Å), types of A-X bonds, the H-H (C-C) bond lengths (Å), X···σ distances (Å) and α and β angles (degrees) are given (see Scheme 3 in the main article text), Table S2: The characteristics of interactions in complexes analysed: the basis set superposition errors, BSSE^1^ and BSSE^2^ (in kcal/mol), for E_int_^1^, and E_int_^2^ energies presented in Table 2 (main article), the H-H or C-C length (in Å) being the Lewis base site in complexes analysed, the C-X or X-X bond length (in Å, X = F,Cl,Br,I) involved in halogen bond, the latter bond stretching frequency, ν (cm^-1^), the stretching frequency intensity, I (km/mol), Table S2b: The characteristics of the electron-accepting bonds for isolated Lewis acid units; the C-X or X-X bond length (in Å), the latter bond stretching frequency, ν_0_ (cm^-1^), the stretching frequency intensity, I_0_ (km/mol), Table S3: The characteristics related to the electron charge transfer: the NBO charge of the Lewis base unit (au), Q_Lb_, the energy of σ_HH/CC_→σ_AX_^*^ orbital–orbital interaction (kcal/mol), the sum of all other orbital–orbital interaction energies (kcal/mol), ΣE_NBO_, the electron density at X⋯σ (or X⋯H/C) BCP (in au), ρ_BCP_, Description of tables and figures, Supplementary Information references.

## Sample Availability

Samples of the compounds are not available from the author.
