# Supplementary material for: A–X⋯σ Interactions—Halogen Bonds with σ-Electrons as the Lewis Base Centre
Source: Molecules. 2021 Aug 26;26(17):5175. doi: 10.3390/molecules26175175 (PMC8434224; doi:10.3390/molecules26175175)
Supplement: Supplementary file 1 [file molecules-26-05175-s001.zip › molecules-1278810-supplementary.pdf]

Supplementary Information

A-X···σ Interactions - Halogen Bonds with σ-Electrons as the Lewis Base Centre

Sławomir J. Grabowski [1,2,*]

[1] Polimero eta Material Aurreratuak: Fisika, Kimika eta Teknologia, Kimika Fakultatea, Euskal Herriko Unibertsitatea UPV/EHU & Donostia International Physics Center (DIPC) PK 1072, 20080 Donostia, Euskadi, Spain
[2] IKERBASQUE, Basque Foundation for Science, 48011 Bilbao, Spain
* s.grabowski@ikerbasque.org

Description of tables and figures

Table S1 collects crystal structures described in the main article that result from CSD searches.

Table S2 contains the C-X and X-X bond stretching frequencies and the corresponding intensities; **Table S2b** presents the characteristics of the electron acepting bonds for isolated Lewis acid units. These bonds participate directly in formation of halogen bonds with σ-bond electrons. It is often claimed in numerous studies that in the case of the A-H···B hydrogen bond the most often occurring effect resulting from its formation is the elongation of the proton donating A-H bond and consequently the shift of the corresponding stretching frequency to lower value (red-shift) [1,2]. However sometimes the shortening of this bond is observed as a result of hydrogen bond formation with the corresponding blue-shift [3-7]. The latter effect is rather rare comparing to the A-H bond elongation. Similarly both cases of the elongation and of the shortening of the A-X bond in A-X···B halogen bonds were described in various studies [8,9]. However the blue-shift occurs here more often than in hydrogen bonds.

In the case of complexes analysed here the elongation of the A-X bond (C-X or X-X) is observed, only in few cases it is its shortening but rather meaningless. The values of %Δr are defined below by eq.1. This term expresses the percentage elongation of the bond in the halogen bonded system (r_{AX}) (Table S2) in relation to its length in isolated Lewis acid unit not involved in any interaction (r_{AX}^{0}) (Table S2b).

$$\%\Delta r = [\frac{(r_{AX} - r_{AX}^{0})}{r_{AX}^{0}}]\ 100\% \qquad (1)$$

It is worth to mention that the negative %Δr values correspond to percentage shortening of the bond (blue-shift). The expression, that is similar to eq.1, may be constructed for the stretching frequency shift resulting from the halogen bond formation. This expression is presented below (eq. 2).

$$\%\Delta \nu = [\frac{(\nu_{AX}^{0} - \nu_{AX})}{\nu_{AX}^{0}}]\ 100\% \qquad (2)$$

The ν_{AX} and ν_{AX}^{0} values correspond to the stretching frequency of the AX bond in the complex and in the isolated Lewis acid unit, respectively. The %Δν value is positive in the case of red-shift and it is negative for the blue-shift. It is interesting that the blue-shifts in these halogen bonded species are observed only in a case of the FCCH complexes where the fluorine acts as the Lewis acid centre. It is well known that the F-atom is the weakest Lewis acid centre if compared

to other halogens. Thus for complexes with blue-shift analysed here the halogen bond is very weak, even for the C_5H_{10}-FCCH and C_5H_{10}-F_2 complexes this interaction does not occur.

Figure S1 presents the relationship between the %Δr and %Δν values if only complexes of cyclopropane are taken into account; the linear correlation is good since R^2 = 0.955. This correlation is good since probably the more pronounced type of interactions are observed for the cyclopropane complexes. These are the A-X···σ interactions where the C-X or X-X bond is clearly directed towards the σ-electrons of the C-C bond. It was pointed out in earlier studies that the accumulation of the electron charge occurs in external part of the C-C bond [10,11] that makes the cyclopropane species similar in properties to unsaturated hydrocarbons. If all complexes are taken into account the correlation is worse.

Table S3 shows the characteristics related to the electron charge transfer.

Figure S2 shows the correlation between the repulsion term, ΔE_{Pauli} and the sum of all attractive terms $\Delta E_{elstat} + \Delta E_{orb} + \Delta E_{disp}$; R^2 is equal to 0.9597 for a linear correlation concerning complexes collected in Table 4 of the main text (the main article). This well correlation is observed in spite of different halogen Lewis acid centres in the sample of complexes analysed. Fig. S2 shows that the orbital energy term, ΔE_{orb}, is well correlated with the repulsion term, ΔE_{Pauli}, R^2 = 0.9453. The linear correlation between the electrostatic term, ΔE_{elstat}, and the repulsion is not so good, R^2 = 0.9135 while the dispersion attraction does not correlate with the repulsion, R^2 = 0.0661.

These correlations (Fig. S2) show that in spite of the fact that for almost all complexes the dispersion term is the most important attractive term the increase of the strength of interaction is connected with the increase of the repulsion that correlates with orbital and electrostatic interaction energy terms. Besides, one can see that the orbital energy related to the electron charge shifts is only one of attractive components, and additionally not the most important term. This is the reason why the charges, overlap energies and electron densities at BCPs, also related to charge shifts, are so small.

Table S1. The crystal structures that contain A-X···σ halogen bonds; refcodes, maximum e.s.d´s values (Å), types of A-X bonds, the H-H (C-C) bond lengths (Å), X···σ distances (Å) and α and β angles (degrees) are given (see Scheme 3 in the main article text).

**Refcode**	**max e.s.d.**	**A-X**	**H-H (C-C)**	**X···σ**	**α**	**β**
Dihydrogen as an electron donor						
NOLJIH	0.01	B-F	1.014	2.432	86.4	113.7
NOLJIH01	0.01	B-F	0.786	2.539	83.8	125.7
NOLJIH02	0.01	B-F	1.015	2.432	86.4	113.6
XAXMIT	0.01	B-F	1.074	2.467	87.6	111.6
Cyclopropane ring as an electron donor						
HEDQAL	0.005	C-F	1.490	2.941	85.3	149.5
HEDQIT	0.005	C-F	1.489	2.940	85.3	149.5
HIGNAO02	0.03	Al-Br	1.388	3.410	87.5	145.5
KIKLIA	0.01	C-Br	1.471	3.332	89.6	159.7
OMIREI	0.03	C-Br	1.508	3.430	89.4	157.1
PILSEK	0.005	C-F	1.499	3.038	89.9	127.1
QUSREC	0.01	C-F	1.489	3.038	89.9	148.6
RUNQEW	0.005	C-Cl	1.524	3.262	88.7	150.1
SUTVOU	0.005	C-Br	1.504	3.372	89.2	165.9
UFIYEP	0.005	C-F	1.527	2.982	87.0	126.4
Cyclobutane ring as an electron donor						
DEZDUH	0.01	C-F	1.565	3.025	89.9	133.5
EQIXAF	0.03	P-F	1.556	3.012	89.9	123.8
EQIXIN	0.03	P-F	1.558	2.989	89.9	125.5

GEGDUU	0.01	C-F	1.521	3.054	89.6	159.7
GOGXOP	0.005	C-F	1.571	2.993	88.4	147.7
HEXYIV	0.005	C-F	1.572	2.971	89.4	122.7
PUFSOA	0.005	C-F	1.548	3.011	89.6	155.8
QASKAX	0.03	Pt-I	1.568	3.551	89.1	189.4
QUBGOJ	0.01	B-F	1.558	3.012	90.0	165.4
SITZOK	0.005	C-F	1.553	3.022	89.8	153.5
TOHDAY	0.005	C-Cl	1.561	3.265	89.1	165.1
TORPOX	0.005	C-F	1.543	3.004	89.1	146.5
YAMGAU01	0.005	C-F	1.562	3.001	88.6	151.0
Cyclopentane ring as an electron donor						
XACHOC	0.005	C-F	1.513	3.020	89.3	153.2
HAFQEN	0.005	C-F	1.544	3.024	89.6	149.4
NARBEQ	0.005	C-Cl	1.505	3.364	89.9	170.9
VANNUT	0.01	B-F	1.603	3.036	90.0	148.0

Table S2. The characteristics of interactions in complexes analysed: the basis set superposition errors, $BSSE^1$ and $BSSE^2$ (in kcal/mol), for E_{int}^1, and E_{int}^2 energies presented in Table 2 (main article), the H-H or C-C length (in Å) being the Lewis base site in complexes analysed, the C-X or X-X bond length (in Å, X=F,Cl,Br,I) involved in halogen bond, the latter bond stretching frequency, ν (cm^{-1}) and the stretching frequency intensity, I (km/mol).

Complex	$BSSE^1$	$BSSE^2$	H-H(C-C)	C-X (X-X)	ν	I
H_2-FCCH	0.02	0.05	0.7331	1.271	1105.45	88.35
H_2-ClCCH	0.01	0.07	0.7434	1.640	761.91	10.01
H_2-BrCCH	0.03	0.25	0.7438	1.791	615.3	0.76
H_2-ICCH	0.05	0.39	0.7446	1.994	529.04	2.23
H_2-F_2	0.02	0.07	0.7432	1.377	1097.98	0.03
H_2-Cl_2	0.03	0.13	0.7439	1.999	585.07	0.12
H_2-Br_2	0.03	0.44	0.7445	2.283	340.11	0.12
H_2-I_2	0.03	0.61	0.7449	2.672	226.98	0.11
C_3H_6-FCCH	0.09	0.24	1.4996	1.270	1107.03	92.04
C_3H_6-ClCCH	0.08	0.35	1.5029	1.640	759.92	7.73
C_3H_6-BrCCH	0.1	1.06	1.505	1.792	612.5	0.07
C_3H_6-ICCH	0.15	1.48	1.5076	1.997	525.52	5.5
C_3H_6-F_2	0.13	0.35	1.5016	1.379	1090.34	1.58
C_3H_6-Cl_2	0.12	0.56	1.5063	2.002	578.76	3.96
C_3H_6-Br_2	0.12	1.69	1.5084	2.287	336.33	2.5
C_3H_6-I_2	0.11	2.15	1.5095	2.675	225.24	1.63
C_4H_8-FCCH	0.11	0.31	1.545	1.271	1106.4	100.72
C_4H_8-ClCCH	0.07	0.36	1.5464	1.639	761.61	9.47
C_4H_8-BrCCH	0.1	1.11	1.5472	1.791	615.1	0.34
C_4H_8-ICCH	0.14	1.57	1.548	1.994	528.94	3.45
C_4H_8-F_2	0.12	0.35	1.5453	1.378	1097.07	0.28
C_4H_8-Cl_2	0.13	0.56	1.5467	1.999	585.51	0.65
C_4H_8-Br_2	0.13	1.71	1.5477	2.283	340.61	0.4
C_4H_8-I_2	0.11	2.23	1.5479	2.672	227.54	0.38
C_5H_{10}-ClCCH	0.07	0.39	1.5503	1.639	761.55	9.64
C_5H_{10}-BrCCH	0.11	1.23	1.5504	1.791	615	0.28
C_5H_{10}-ICCH	0.15	1.71	1.5504	1.995	527.45	3.92

C_5H_{10}-Cl_2	0.13	0.62	1.5483	2.000	585.24	0.96
C_5H_{10}-Br_2	0.14	1.85	1.5475	2.284	339.96	0.68
C_5H_{10}-I_2	0.12	2.41	1.5504	2.672	227.78	0.62

Table S2b. The characteristics of the electron acepting bonds for isolated Lewis acid units; the C-X or X-X bond length (in Å), the latter bond stretching frequency, ν_0 (cm^{-1}) and the stretching frequency intensity, I_0 (km/mol).

C-X (X-X)	ν_0	I_0
1.271	1105.19	86.5
1.639	762.63	10.51
1.790	616.4	1.12
1.994	530.47	1.37
1.377	1098.59	0
1.998	586.68	0
2.281	342.33	0
2.669	228.56	0

Table S3. The characteristics related to the electron charge transfer: the NBO charge of the Lewis base unit (au), Q_{Lb}, the energy of $\sigma_{HH/CC}\rightarrow\sigma_{AX}^*$ orbital-orbital interaction (kcal/mol), the sum of all other orbital-orbital interaction energies (kcal/mol), ΣE_{NBO}, the electron density at X···σ (or X···H/C) BCP (in au), ρ_{BCP}.

Complex	Q_{Lb}	$\sigma_{HH/CC}\rightarrow\sigma_{AX}^*$	ΣE_{NBO}	ρ_{BCP}
H_2-FCCH	0.000	0	0	0.001
H_2-ClCCH	0.001	0.11	0	0.002
H_2-BrCCH	0.001	0.29	0	0.004
H_2-ICCH	0.003	0.57	0	0.006
H_2-F_2	0.001	0.06	0	0.001
H_2-Cl_2	0.003	0.34	0	0.004
H_2-Br_2	0.005	0.55	0	0.006
H_2-I_2	0.006	0.1	0	0.006
C_3H_6-FCCH	0.000	0	0	0.002
C_3H_6-ClCCH	0.002	0.06	0	0.005
C_3H_6-BrCCH	0.005	0.12	0	0.006
C_3H_6-ICCH	0.008	0.26	0.58	0.007
C_3H_6-F_2	0.012	0.07	0	0.004
C_3H_6-Cl_2	0.019	0.29	0.46	0.007
C_3H_6-Br_2	0.025	0.41	0.55	0.008
C_3H_6-I_2	0.022	0.37	0.51	0.008

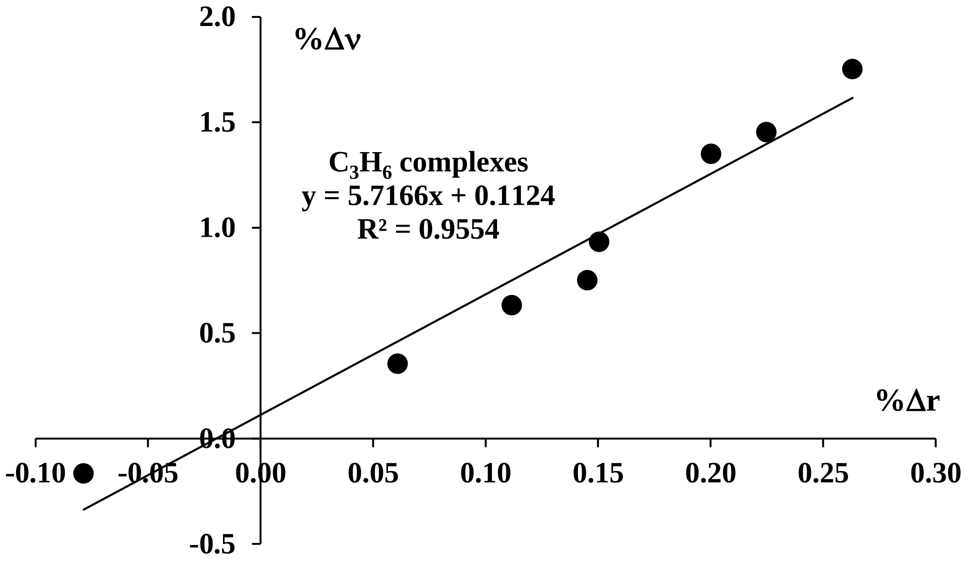

Figure S1. The dependence between %Δr and %Δν, the correlation for cyclopropane complexes is shown (black circles).

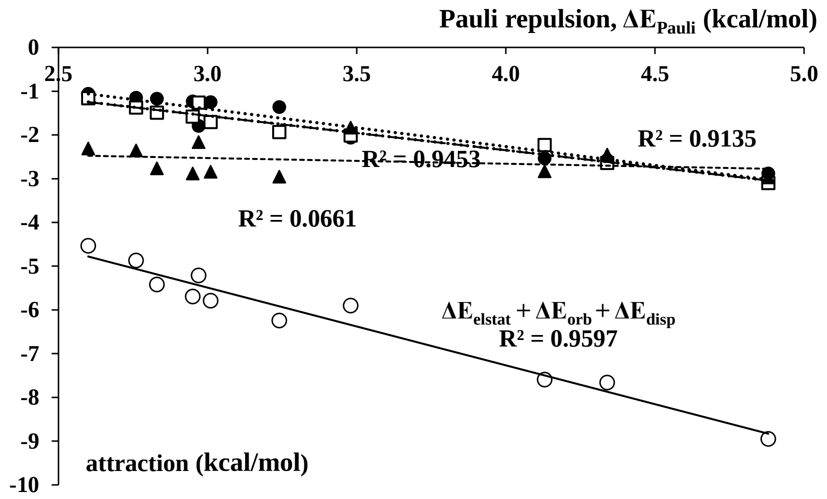

Figure S2. The relationship between the sum of attractive interaction energy terms and the Pauli repulsion term (white circles). Relationships between the Pauli repulsion and separated attractive terms are presented; orbital (white squares), electrostatic (black circles), dispersion (black triangles).

Supplementary Information references

1. Reed, E.; Curtiss, L.A.; Weinhold, F. Intermolecular interactions from a natural bond orbital, donor-acceptor viewpoint. *Chem. Rev.* **1988**, *88*, 899–926.
2. Weinhold, F.; Landis, C. *Valency and Bonding, a Natural Bond Orbital Donor—Acceptor Perspective*; Cambridge University Press: Cambridge, UK, 2005.

3. Pinchas, S. Infrared Absorption of Aldehydic C-H Group. *Analitical Chemistry* **1955**, *27*, 2-6.
4. Pinchas, S. Infrared Absorption of Aldehydic C-H Group. *Analitical Chemistry* **1957**, *29*, 334-339.
5. Hobza, P.; Havlas, Z. Blue-shifting hydrogen bonds. *Chem. Rev.* **2000**, *100*, 4253-4264.
6. Gu, Y.; Kar, T.; Scheiner, S. Fundamental Properties of the CH···O Interaction: Is It a True Hydrogen Bond? *J. Am. Chem. Soc*. 1999, **121**, 9411-9422.
7. Kryachko, E.S. *Neutral Blue-Shifting and Blue-Shifted Hydrogen Bonds*, pages 293-336, Chapter 8 in the book *Hydrogen Bonding – New Insights*, Ed. Grabowski, S.J. Springer, 2006.
8. Zhou, J.-W.; Jiang, Y.-J.; Guo, M.; Hu, G.-X.; Zhang, B.; Liu, H.-C.; Yu, Q.-S. Ab Initio Study of the Complexes of Halogen-Containing Molecules RX (X=Cl, Br, and I) and NH_3: Towards Understanding the Nature of Halogen Bonding and the Electron-Accepting Propensities of Covalently Bonded Halogen Atoms. *Chem. Eur. J.* **2005**, *11*, 740-751.
9. Zierkiewicz, W.; Bieńko, D.C.; Michalska, D.; Zeegers-Huyskens, T. Theoretical Investigation of the Halogen Bonded Complexes Between Carbonyl Bases and Molecular Chlorine. *J. Comput. Chem*. **2015**, *36*, 821–832.
10. Galano, A.; Alvarez-Idaboy, J.R.; Vivier-Bunge, A. Non-alkane behavior of cyclopropane and its derivatives: characterization of unconventional hydrogen bond interactions. *Theor. Chem. Acc.* **2007**, *118*, 597–606 and references therein.
11. Rozas, I.; Alkorta, I.; Elguero, J. Unusual Hydrogen Bonds: H···π Interactions. *J. Phys. Chem. A* **1997**, *101*, 9457–9463.
